# Determination of method-specific reference intervals for vitamin B12 and its correlation with holotranscobalamin

**DOI:** 10.11613/BM.2026.030705

**Published:** 2026-10-15

**Authors:** Ozlem C. Madenci, Ebru Ertug, Nazire Aladag, Ferruh K. Isman, Ibrahim Yılmaz

**Affiliations:** 1 Department of Clinical Biochemistry, Kartal Dr Lutfi Kirdar City Hospital, Istanbul, Turkey; 2 Department of Internal Medicine, Kartal Dr Lutfi Kirdar City Hospital, Istanbul, Turkey; 3 Department of Clinical Biochemistry, Medeniyet University, Istanbul, Turkey; 4 Department of Clinical Biochemistry, Haseki Training and Research Hospital, Istanbul, Turkey

**Keywords:** reference ranges, transcobalamins, vitamin B 12, vitamin B 12 deficiency

## Abstract

**Introduction:**

We aimed to compare total serum vitamin B12 measurements across four immunoassay platforms, establish method-specific reference intervals, evaluate the clinical impact of a uniform deficiency cut-off, and assess associations between total vitamin B12 and holotranscobalamin (holoTC).

**Materials and methods:**

Residual serum samples from 220 apparently healthy adults undergoing routine health check-ups were analyzed. Total vitamin B12 was measured on Beckman, Abbott, Siemens, and Roche platforms. Beckman analyses used the updated Access Vitamin B12 II calibrator introduced in December 2024. Holotranscobalamin was measured on the Abbott system. Reference intervals were derived using non-parametric methods according to CLSI EP28-A3c guidelines. Method comparison was performed using Passing-Bablok regression and concordance correlation coefficients (CCC), and associations between total vitamin B12 and holoTC were assessed using Spearman correlation analysis.

**Results:**

Total vitamin B12 concentrations differed significantly across platforms, with moderate to good agreement (CCC: 0.81-0.91). Study-derived lower reference limits (LRLs) varied substantially between assays and differed from manufacturer-provided values, most prominently for the Beckman platform. Application of a fixed deficiency cut-off of 148 pmol/L resulted in marked method-dependent differences in deficiency classification. Correlations between total vitamin B12 and holoTC were low to moderate across analytical platforms.

**Conclusions:**

Serum vitamin B12 results vary across platforms, causing method-dependent LRLs and inconsistent classification with uniform cut-offs, underscoring the need for method-specific interpretation and improved harmonization. Low-to-moderate correlations reflect that holoTC and total vitamin B12 represent different components of vitamin B12 status and that total vitamin B12 assays differ by platform in reflecting the active fraction.

## Graphical abstract

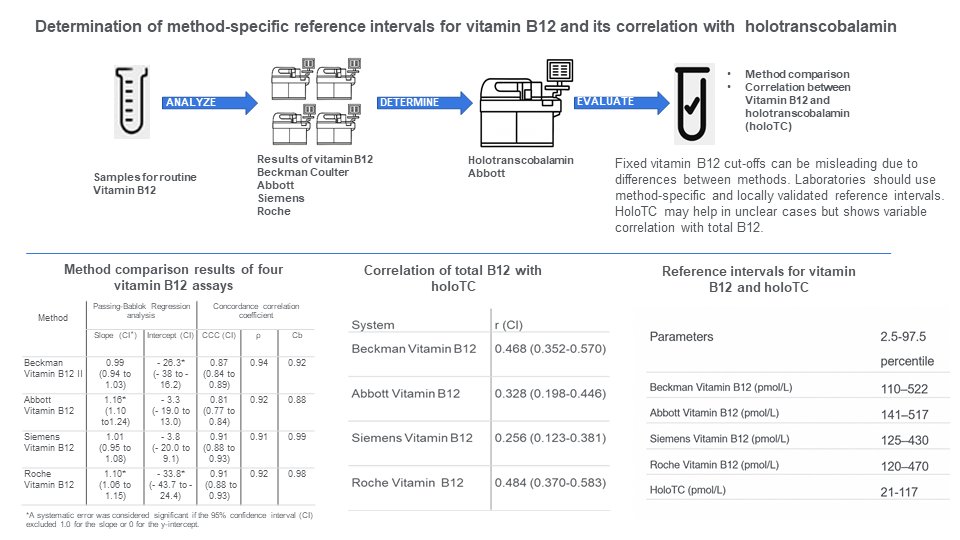

## Highlights

• Fixed vitamin B12 cut-offs can misclassify deficiency due to method differences

• Laboratories should use method-specific and locally validated reference intervals

• Holotranscobalamin shows low to moderate correlation with total B12

## Introduction

Vitamin B12 is an essential micronutrient involved in DNA synthesis, erythropoiesis, and neurological function, and its deficiency may lead to clinically significant conditions such as megaloblastic anemia, peripheral neuropathy, and cognitive impairment (*1*). Accurate laboratory assessment of vitamin B12 status is therefore critical for appropriate diagnosis and patient management. However, there is currently no universally accepted gold standard test for diagnosing vitamin B12 deficiency, and available laboratory assays primarily serve as supportive tools rather than definitive tests for diagnosis (*2*).

In routine clinical practice, total serum vitamin B12 remains the most widely used first-line test owing to its analytical feasibility, compatibility with automated platforms, and cost-effectiveness, while biomarkers such as methylmalonic acid (MMA) and holotranscobalamin (holoTC) are mainly used as second-line or complementary tests (*2*-*4*).

In circulation, approximately 80% of vitamin B12 is bound to haptocorrin and is biologically inactive, whereas only the transcobalamin-bound fraction (holoTC), accounting for about 20% of total circulating vitamin B12, is available for cellular uptake. Consequently, total serum vitamin B12 concentrations may not reliably reflect early or functional deficiency. In this context, holoTC has been proposed as a more sensitive and specific biomarker in selected clinical settings, particularly among individuals with borderline vitamin B12 concentrations, older adults, pregnant women, and patients with impaired absorption (*e.g*., post-gastrectomy). Nevertheless, the routine use of holoTC as a first-line test remains limited due to higher costs and insufficient evidence supporting its cost-effectiveness (*2*, *4*-*6*).

Consequently, total serum vitamin B12 measurement remains the mainstay of laboratory assessment. However, its analytical determination is inherently challenging due to low circulating concentrations, complex binding characteristics, and the absence of a fully harmonized reference measurement procedure (*2*). Although the introduction of the WHO International Reference Reagent (IRR 03/178) marked an important step toward standardization, substantial inter-assay variability persists among commercially available immunoassays, primarily driven by differences in calibration, antibody specificity, and assay design, limiting the comparability of results across platforms (*1*, *7*, *8*). Moreover, traceability to the WHO International Standard (IS 03/178) has not been fully implemented across all commercially available total vitamin B12 immunoassays. Comparative studies evaluating the performance of current total vitamin B12 assays using patient serum samples remain limited (*9*, *10*).

In addition to analytical variability, there is no universal consensus on clinical decision thresholds for vitamin B12 deficiency. National Institute for Health and Care Excellence (NICE) defines total vitamin B12 concentrations < 133 pmol/L as deficiency and 133-258 pmol/L as possible deficiency requiring confirmatory testing, while slightly higher thresholds (148 pmol/L) have also been applied in clinical practice (*2*, *11*, *12*). National Institute for Health and Care Excellence permits the use of locally validated lower reference limits (LRLs) when substantial method-related variability is present (*2*). Accordingly, guideline-recommended diagnostic cut-offs should be applied only when an assay’s LRL is broadly comparable; otherwise, result interpretation should rely on assay-specific or locally validated reference limits with careful clinical judgment. Fixed diagnostic cut-offs fail to account for method-dependent analytical differences and may therefore result in inconsistent clinical classification across platforms. Although manufacturer-reported LRLs vary across the evaluated assays, Beckman Coulter applies the highest manufacturer-reported lower reference limit, highlighting persistent inter-assay variability (*7*, *8*). However, comparative data evaluating the impact of platform-specific reference intervals on clinical classification in routine laboratory practice remain limited. Moreover, reference intervals derived from healthy populations reflect statistical distributions rather than functional vitamin B12 sufficiency and may miss early or subclinical deficiency (*1*, *2*, *6*).

In December 2024, Beckman Coulter introduced the Access Vitamin B12 II Calibrators (REF D06116), traceable to internal standards aligned with the WHO IRR 03/178 in accordance with EN ISO 17511. Although an average recovery of 111% relative to the WHO-assigned concentration of 354 pmol/L has been reported, suggesting improved analytical alignment, the extent to which this recalibration improves inter-platform clinical comparability has not yet been established (*13*). It therefore remains uncertain whether such recalibration efforts are sufficient to enable uniform application of vitamin B12 deficiency cut-offs across immunoassay platforms, or whether method-specific analytical characteristics continue to influence LRLs and deficiency classification.

We hypothesized that total serum vitamin B12 measurements differ across commonly used immunoassay platforms (Beckman, Abbott, Siemens, and Roche), resulting in method-specific reference intervals and platform-dependent differences in clinical classification when a uniform deficiency cut-off is applied. We further hypothesized that correlations between total vitamin B12 and holoTC vary across analytical platforms.

## Materials and methods

This retrospective study was conducted in the Biochemistry Laboratory between February 1 and June 1, 2025. A total of 220 adults (≥ 18 years) attending the internal medicine outpatient clinic for routine health check-ups were included. Clinical records and laboratory data were reviewed in collaboration with an internal medicine specialist. Individuals with conditions known to affect vitamin B12 status were excluded, including chronic systemic or gastrointestinal diseases, malignancy, pregnancy or lactation, and vitamin supplementation within the preceding 3 months. Patients using medications known to interfere with vitamin B12 metabolism, such as metformin, proton pump inhibitors, H2-receptor antagonists, antiepileptic drugs, or those with a history of nitrous oxide exposure were also excluded, as were individuals with clinical or laboratory findings that could confound the assessment of vitamin B12 status (*1*). Laboratory-based exclusion criteria included anemia (hemoglobin < 120 g/L for women and < 130 g/L for men), macrocytosis (mean corpuscular volume > 100 fL), depleted iron stores (serum ferritin < 15 µg/L), and folate deficiency (serum folate < 6.8 nmol/L) (*14*-*17*).

Fasting venous blood samples were collected in serum separator tubes (SST) (Vacusera, Disera A.Ş., Izmir, Turkey; lot no. 235305) and centrifuged within 1 hour at 3000xg for 10 minutes. At the end of each day, residual serum samples from eligible individuals were identified, aliquoted, and stored at - 80 °C for up to two months under controlled conditions to ensure sample stability prior to analysis. All measurements were performed on the same thawed aliquots using four automated immunoassay platforms, in accordance with CLSI EP09c guidelines (*18*): Beckman DXi 800 Unicel (REF no. 33000) (Beckman Coulter, Brea, USA), Abbott Alinity i 1000 (REF no. 07P6732) (Abbott Laboratories, Abbott Park, USA), Siemens Atellica IM Analyzer (REF no. 57258298) (Siemens Healthineers, Erlangen, Germany), and Roche Cobas e801 (REF no. 806567702) (Roche Diagnostics, Basel, Switzerland). Holotranscobalamin measurements were performed simultaneously on the Abbott Alinity i 1000 system (REF no. 10995088).

All platforms employ competitive assay designs with chemical pretreatment to release vitamin B12, generating signals inversely proportional to analyte concentration. Intrinsic factor-mediated binding is a common feature of all assays; however, methodological differences exist in signal detection. The Abbott Alinity i 1000, Siemens ADVIA Centaur XP/Atellica IM, and Roche Cobas e801 assays are intrinsic factor-based protein-binding methods without direct antibody recognition of vitamin B12, using chemiluminescent microparticle immunoassay (CMIA), direct chemiluminescent immunoassay (CLIA), and electrochemiluminescence immunoassay (ECLIA) principles, respectively. In contrast, the Beckman Coulter Access assay is a competitive immunoenzymatic method in which vitamin B12 binds to an intrinsic factor-enzyme conjugate and detection is achieved through antibody-mediated capture of intrinsic factor-bound complexes.

As this study was retrospective and based on existing laboratory results and residual serum samples, no additional intervention was performed and individual informed consent was not required. The study was approved by the institutional ethics committee of Dr. Lütfi Kırdar Kartal City Hospital (Decision No: 2025/010.99/18/13.

### Statistical analysis

Data distribution was assessed using the Kolmogorov-Smirnov test. As the data were non-normally distributed, results are presented as the median and interquartile range (IQR). Reference intervals were calculated using the non-parametric quantile method, defined by the 2.5th and 97.5th percentiles of values obtained from the healthy population, together with 90% confidence intervals (CI), in accordance with CLSI EP28-A3c guidelines (*19*).

Outliers were identified using the Tukey fence method. Sex-related differences were evaluated using the standard normal deviation (z) test, with calculated z-values compared against the critical z-value. Age-related partitioning was assessed by stratifying participants into four age groups (18-39, 40-59, 60-69, and ≥ 70 years), and differences in vitamin B12 concentrations among age groups were analyzed using the Kruskal-Wallis test (*20*).

Method comparison was performed using Passing-Bablok regression analysis and the CCC. Comparisons were based on the mean vitamin B12 concentration derived from the four analytical platforms, as no single reference measurement procedure was available. Proportional systematic error was considered significant when the 95% confidence interval for the slope did not include 1.0, and constant systematic error when the 95% CI for the intercept did not include 0.

The relationship between holoTC and total vitamin B12 concentrations measured across platforms was evaluated using Spearman correlation analysis. All statistical analyses were performed using MedCalc Statistical Software version 12 (MedCalc Software, Mariakerke, Belgium).

## Results

Analytical performance characteristics of the four vitamin B12 assays, based on manufacturer specifications, are summarized in Table 1. Demographic characteristics and laboratory parameters used for population characterization and vitamin B12 assessment are presented in Table 2, while reference interval data for total vitamin B12 and holoTC are shown in Table 3. Initially, 225 apparently healthy individuals were included for reference interval analysis; five were identified as outliers and excluded, yielding a final study population of 220 individuals.

**Table 1:** Analytical performance characteristics of vitamin B12 assays by manufacturer claims

	**Beckman** **Vitamin B12**	**Abbott** **Vitamin B12**	**Siemens** **Vitamin B12**	**Roche** **Vitamin B12**
Test name	B12 II	Alinity i B12	Atellica IM B12	Elecsys B12 II
Imprecision across measuring range (total CV %)	< 12%	< 7.9%	< 10%	< 8.1%
Analytical sensitivity (pmol/L)	LoB < 58 LoD < 77 LoQ < 77	LoB < 61 LoD < 80 LoQ < 109	LoB < 33 LoD < 66 LoQ < 66	LoB < 37 LoD < 74 LoQ < 111
Linearity (pmol/L)	75-1549	109-1476	33-1476	74-1476
Reference intervals (pmol/L)	164-1062	138-652	156-672	145-569
CV - coefficient of variation. LoB - limit of blank. LoD - limit of detection. LoQ - limit of quantification.				

**Table 2:** Demographic and laboratory characteristics of the study population

**Parameters**	**Median** **(N = 220)**	**2.5-97.5th** **percentile**
Age (median, range) (years)	43 (18-89)	18-75
Sex, N (%)		Female 128 (58%) Male 92 (42%)
Hemoglobin (g/L)	135	128-145
Hematocrit (L/L)	0.399	0.298-0.493
MCV (fL)	85.7	61.67-95.72
Folate (nmol/L)	16.61	7.79-38.93
Ferritin (µg/L)	218	23.9-1341
MCV - mean corpuscular volume.		

**Table 3:** Reference intervals for vitamin B12 and holotranscobalamin

**Parameters**	**Median** **(N = 220)**	**2.5-97.5th** **percentile**	**Lower limit** **90% CI**	**Upper limit** **90% CI**
Beckman Vitamin B12 (pmol/L)	203	110–522	95-118	413-784
Abbott Vitamin B12 (pmol/L)	278	141–517	124-156	452-579
Siemens Vitamin B12 (pmol/L)	234	125–430	97-137	407-472
Roche Vitamin B12 (pmol/L)	227	120–470	101-128	401-523
HoloTC (pmol/L)	53	21-117	19-23	108-120
HoloTC - holotranscobalamin. CI - confidence interval.				

Sex-based partitioning was evaluated using the Harris-Boyd Z test. The calculated Z values for Beckman, Abbott Alinity, Siemens, and Roche assays (1.25, 1.39, 2.27, and 0.96, respectively) were all below the critical value (Z* = 2.87); therefore, sex-specific reference intervals were not warranted, and combined reference intervals were applied across all platforms. Age-related differences in vitamin B12 concentrations were assessed using the Kruskal-Wallis test and were not statistically significant for any platform (Beckman, P = 0.103; Abbott, P = 0.060; Siemens, P = 0.743; Roche, P = 0.136). Accordingly, age-specific reference intervals were not established.

Passing-Bablok regression analyses comparing each analytical platform with the mean vitamin B12 concentration are provided in Supplementary Figure 1. Concordance correlation coefficients demonstrated moderate to good agreement between individual platforms and the reference mean, with values ranging from 0.81 to 0.91 (Table 4).

**Table 4:** Method comparison results of four vitamin B12 assays

	**Passing-Bablok regression analysis**		**Concordance correlation coefficient**		
**Method**	Slope (CI)*	**Intercept (CI)**	**CCC (CI)**	**ρ**	**^C^b**
Beckman Vitamin B12 II	0.99 (0.94 to 1.03)	- 26.3* (- 38.0 to - 16.2)	0.87 (0.84 to 0.89)	0.94	0.92
Abbott Vitamin B12	1.16* (1.10 to 1.24)	- 3.3 (- 19.0 to 13.0)	0.81 (0.77 to 0.84)	0.92	0.88
Siemens Vitamin B12	1.01 (0.95 to 1.08)	- 3.8 (- 20.0 to 9.1)	0.91 (0.88 to 0.93)	0.91	0.99
Roche Vitamin B12	1.10* (1.06 to 1.15)	- 33.8* (- 43.7 to - 24.4)	0.91 (0.88 to 0.93)	0.92	0.98
Comparisons were based on the mean vitamin B12 concentration across four platforms. *A systematic error was considered significant if the 95% confidence interval excluded 1.0 for the slope or 0 for the y-intercept. CCC - concordance correlation coefficient. CI - confidence interval. ρ - Pearson correlation coefficient. Cb - bias correction factor.					

Method-specific reference intervals for serum vitamin B12 were established and compared with manufacturer-reported limits. The study-derived LRLs were lower than the manufacturer-provided values for Beckman (110 *vs.* 164 pmol/L), Roche (120 *vs.* 145 pmol/L), and Siemens (125 *vs.* 156 pmol/L), whereas Abbott was the only platform for which the study-derived LRL exceeded the manufacturer’s reported value (141 *vs.* 138 pmol/L).

Using manufacturer-provided reference intervals, the proportion of individuals classified as having low vitamin B12 concentrations differed markedly between platforms, being highest for Beckman (29.1%), intermediate for Roche (8.6%) and Siemens (8.2%), and lowest for Abbott (1.8%).

When a uniform deficiency threshold of 148 pmol/L was applied, the proportion of samples below the cut-off varied across platforms, ranging from 2.7% for Abbott to 15.0% for Beckman, with intermediate rates for Siemens (6.8%) and Roche (9.5%) (*11*, *12*). Spearman’s correlation analysis showed that the association between total vitamin B12 and holoTC was moderate for Roche (r = 0.48) and Beckman Coulter (r = 0.47), and low for Abbott (r = 0.33) and Siemens (r = 0.26) (Table 5).

**Table 5:** Correlation of total B12 with holotranscobalamin

**System**	**r (CI)**
Beckman Vitamin B12	0.47 (0.35-0.57)
Abbott Vitamin B12	0.33 (0.20-0.45)
Siemens Vitamin B12	0.26 (0.12-0.38)
Roche Vitamin B12	0.48 (0.37-0.58)
r - Spearman correlation coefficient. CI - confidence interval.	

## Discussion

Persistent inter-assay variability and incomplete harmonization limit the clinical comparability of total vitamin B12 results, while uncertainty over whether clinical classification should be based on fixed diagnostic cut-offs, manufacturer-provided reference intervals, or locally derived reference limits further complicates result interpretation. In line with these challenges, method comparison analyses in the present study indicate that total vitamin B12 measurements are not fully interchangeable across analytical platforms. Although concordance analysis indicated overall moderate to good agreement with the reference mean, Passing-Bablok regression revealed assay-dependent systematic differences, with Beckman showing a constant negative bias, Abbott exhibiting proportional bias, Roche showing both proportional and constant negative bias, whereas Siemens showed minimal systematic bias. Differences in assay design and detection principles (CLIA, CMIA, and ECLIA) may underlie the observed inter-assay variability and should be considered when interpreting vitamin B12 results, particularly when fixed diagnostic cut-offs are applied or results are compared across platforms. These findings are consistent with previous reports demonstrating assay-dependent variability in vitamin B12 measurement. İspir *et al.* evaluated serum vitamin B12 concentrations using four automated immunoassays-Beckman Coulter DxI 800 UniCel, Siemens ADVIA Centaur XP, Roche Cobas e801, and Abbott Architect i2000sr-and reported high overall correlations among methods. The Beckman Coulter DxI 800 UniCel assay yielded lower vitamin B12 concentrations than the other platforms, with a mean bias of - 90 pmol/L, in line with our observation of a constant negative bias for the Beckman assay. Notably, despite these analytical differences, all four methods showed comparable performance in reflecting functional vitamin B12 status, as indicated by similar correlations with methylmalonic acid and homocysteine (*21*).

These assay-dependent analytical differences directly influence the clinical interpretation of vitamin B12 results, particularly when uniform cut-offs are applied across different analytical platforms. In this context, our findings highlight the distinct roles of clinical decision limits and reference intervals, as well as the need to apply them in a method-dependent manner when interpreting vitamin B12 results. For vitamin B12, clinical decision limits are outcome-based values derived from external clinical evidence and define concentrations associated with an increased risk of deficiency, whereas reference intervals are method-dependent and reflect the analytical characteristics of individual assays (*22*).

Analysis of identical samples from our clinically healthy cohort showed that application of a fixed vitamin B12 cut-off (148 pmol/L) resulted in markedly different deficiency classifications across assays, reflecting incomplete inter-assay harmonization. Especially when vitamin B12 concentrations are close to diagnostic decision limits, use of a universal cut-off may lead to inconsistent clinical interpretation across analytical platforms. Assay-dependent differences also persisted when manufacturer-provided reference intervals were applied, with Beckman identifying the highest and Abbott the lowest proportion of results below the lower limit, while Roche and Siemens showed intermediate patterns. This finding indicates a mismatch between manufacturer-defined reference limits and the LRLs observed in our study population. Importantly, method-specific reference intervals should not be regarded as direct substitutes for clinical decision limits, as their isolated use may obscure biochemical deficiency in the absence of appropriate clinical context (*2*).

In line with our findings, Jassam *et al.* reported assay-dependent differences in vitamin B12 reference intervals across Roche, Siemens, and Beckman DXi platforms, with the Beckman assay showing a lower reference limit (81 pmol/L) compared with the others. These differences were attributed to the lack of standardized reference materials and the absence of higher-order reference measurement procedures (*8*). To address whether NICE-recommended vitamin B12 cut-offs can be applied across different analytical platforms, Cesana *et al.* evaluated inter-assay variability in total vitamin B12 measurements, with particular focus on concentrations close to clinical decision thresholds. Agreement among commonly used total vitamin B12 immunoassays was assessed using 19 external quality assessment exercises and 97 serum samples measured on Abbott, Beckman, Siemens, and Roche platforms, with Roche serving as a WHO International Standard–traceable reference. The authors demonstrated substantial inter-method variability, with Abbott consistently yielding higher and Beckman yielding lower vitamin B12 concentrations relative to Roche; notably, Beckman exhibited both proportional and constant systematic errors. These findings challenge the interchangeability of fixed diagnostic thresholds across assays and support the need for assay-aware interpretation of vitamin B12 results (*10*). The NICE-recommended 133 pmol/L threshold for absolute vitamin B12 deficiency is method-dependent and based on earlier analytical methodologies that may not be fully harmonized with contemporary standardized assay platforms, potentially limiting cross-platform comparability. Notably, studies using Abbott-based assays have suggested higher deficiency thresholds (~180 pmol/L), indicating that strict adherence to the 133 pmol/L cut-off may underestimate the prevalence of vitamin B12 deficiency in individuals who could benefit from supplementation (*2*, *9*, *23*). In the study by Cesana *et al.*, Roche traceable to the WHO International Standard was used as the reference method, and regression-based conversion was applied to estimate corresponding cut-off values for other platforms. Using this approach, a markedly lower threshold of approximately 109 pmol/L was estimated for Beckman, and recalibration of the Beckman system was recommended before reliable application of fixed cut-offs. In our study, conducted after implementation of the WHO-traceable Access Vitamin B12 II calibrator (IS 03/178), method-dependent differences in LRLs persisted, with Siemens and Roche showing closely aligned values, Beckman yielding the lowest (110 pmol/L), and Abbott the highest. These findings indicate that inter-assay variability continues to influence vitamin B12 classification even in a clinically healthy cohort, despite recent calibration updates (*7*, *10*).

In a Roche-based reference interval study including over 27,000 individuals, a higher lower reference limit (140 *vs.* 120 pmol/L) was reported compared with our study, likely reflecting population differences and the inability to exclude vitamin B12 supplementation in retrospective designs, underscoring the importance of locally derived, population-based reference intervals (*24*). A study in healthy young adults reported vitamin B12 reference intervals differing from manufacturer-provided ranges and found no gender-related differences, in line with our findings (*25*). National Health and Nutrition Examination Survey (NHANES) analyses indicate that the higher vitamin B12 concentrations observed with increasing age are largely attributable to greater supplement use; accordingly, no age-related variation was observed in our healthy cohort, from which supplement users were excluded (*20*).

According to NICE recommendations, holoTC provides complementary information in selected clinical contexts and should not be used as a substitute for total vitamin B12 measurement (*2*). In our healthy adult cohort, holoTC showed low-to-moderate correlations with total vitamin B12 across all analytical platforms, consistent with previous reports (*1*, *3*). This is expected, as holoTC represents the biologically active fraction of vitamin B12 rather than total circulating concentrations. Accordingly, holoTC and total vitamin B12 assess different aspects of vitamin B12 status and are not interchangeable. The platform-dependent variability in correlation strength further suggests that total vitamin B12 assays differ in how well they reflect the biologically active fraction.

This study evaluated a limited number of immunoassay platforms, while other systems used in different regions were not assessed, which may limit generalizability. Nevertheless, the included assays represent the most widely used and up-to-date platforms available in our laboratory at the time of the study. As none of the evaluated assays constitutes a reference measurement procedure, method comparisons were based on the consensus mean of the four platforms. Due to its retrospective design, only prescribed and documented vitamin B12 supplementation recorded in hospital electronic medical records could be evaluated, whereas undocumented over-the-counter vitamin use could not be completely assessed. Importantly, reference intervals were derived from a carefully selected healthy population, minimizing bias related to vitamin B12 supplementation that cannot be excluded in retrospective studies and thereby enhancing the clinical relevance of the lower reference limits.

In conclusion, this study demonstrates that total serum vitamin B12 concentrations differ substantially across commonly used immunoassay platforms, resulting in method-dependent variation in LRLs and underscoring the need for improved assay harmonization. Application of a uniform deficiency cut-off does not adequately account for these analytical differences and leads to variable deficiency classification, even among apparently healthy individuals. Accordingly, total vitamin B12 cut-offs should not be assumed to be universally applicable across analytical platforms. Accurate assessment of vitamin B12 status requires interpretation beyond fixed cut-offs, incorporating method-specific, locally derived reference limits within the appropriate clinical context. Finally, the low-to-moderate correlations observed indicate that holoTC and total vitamin B12 reflect different components of vitamin B12 status and are not interchangeable, with total vitamin B12 assays differing by platform in their reflection of the biologically active fraction.

## Data Availability

The data generated and analyzed in the presented study are available from the corresponding author on request.
